# Do Nutrient-Based Front-of-Pack Labelling Schemes Support or Undermine Food-Based Dietary Guideline Recommendations? Lessons from the Australian Health Star Rating System

**DOI:** 10.3390/nu10010032

**Published:** 2018-01-05

**Authors:** Mark A. Lawrence, Sarah Dickie, Julie L. Woods

**Affiliations:** Institute for Physical Activity and Nutrition (IPAN), School of Exercise and Nutrition Sciences, Deakin University, Geelong 3220, Australia; disar@deakin.edu.au (S.D.); j.woods@deakin.edu.au (J.L.W.)

**Keywords:** food-based dietary guidelines, front-of-pack labelling, health star rating, nutrient profiling, reductionist, dietary patterns

## Abstract

Food-based Dietary Guidelines (FBDGs) promote healthy dietary patterns. Nutrient-based Front-of-Pack Labelling (NBFOPL) schemes rate the ‘healthiness’ of individual foods. This study aimed to investigate whether the Australian Health Star Rating (HSR) system aligns with the Australian Dietary Guidelines (ADGs). The Mintel Global New Products Database was searched for every new food product displaying a HSR entering the Australian marketplace from 27 June 2014 (HSR system endorsement) until 30 June 2017. Foods were categorised as either a five food group (FFG) food or ‘discretionary’ food in accordance with ADG recommendations. Ten percent (1269/12,108) of new food products displayed a HSR, of which 57% were FFG foods. The median number of ‘health’ stars displayed on discretionary foods (2.5; range: 0.5–5) was significantly lower (*p* < 0.05) than FFG foods (4.0; range: 0.5–5), although a high frequency of anomalies and overlap in the number of stars across the two food categories was observed, with 56.7% of discretionary foods displaying ≥2.5 stars. The HSR system is undermining the ADG recommendations through facilitating the marketing of discretionary foods. Adjusting the HSR’s algorithm might correct certain technical flaws. However, supporting the ADGs requires reform of the HSR’s design to demarcate the food source (FFG versus discretionary food) of a nutrient.

## 1. Introduction

Dietary risk factors are the leading contributors to the global burden of disease [1]. Over the past four to five decades, Food-based Dietary Guidelines (FBDGs) have been developed in more than 100 countries to provide advice to the general public on foods, food groups and dietary patterns to tackle these dietary risk factors, to promote overall health and prevent obesity and diet-related non-communicable diseases (NCDs) [2]. More recently, Nutrient-based Front-of-Pack Labelling (NBFOPL) schemes have become prominent around the world as an approach also intended to help prevent obesity and NCDs [3].

FBDGs and NBFOPL schemes represent evidence-informed manifestations of two alternative paradigms of nutrition science about the causes of and solutions to dietary risk factors. FBDGs operate within a holistic paradigm in which the causes of dietary risk factors are seen to be dietary inadequacies, excesses and imbalances and it is changes to the amount, type and variety of foods within dietary patterns that it is believed are necessary to correct dietary risk factors. Alternatively, NBFOPL schemes operate within a reductionist paradigm in which the causes of dietary risk factors are seen to be nutrient inadequacies and excesses, and it is changes to the amount of nutrients within foods that it is believed are necessary to correct dietary risk factors. Are these two approaches complementary or conflicting in efforts to prevent obesity and NCDs? While reservations have been raised about the sufficiency of a nutrient approach to effectively tackle contemporary nutrition problems [4], in the context of food labelling it has been described as having significant potential [5].

Current developments with FBDGs and a NBFOPL scheme in Australia provide a case study to investigate this dilemma. In 2013, the revised Australian Dietary Guidelines (ADGs) were launched. They state,

*People eat whole foods rather than single nutrients … For this reason, these Guidelines make recommendations based only on whole foods … rather than recommendations related to specific food components and individual nutrients*.

[6] (p. 3)

They advise people to enjoy a wide variety of nutritious Five Food Group (FFG) foods (fruit; vegetables; grain foods; meat/eggs/tofu/nuts/seeds/legumes; milk/yoghurt/cheese/alternatives) every day, and limit intake of non-nutritious ‘discretionary’ foods (and high in kilojoules, saturated fat, added sugars, added salt, or alcohol) [6,7]. Yet, results from the 2011–2012 Australian National Nutrition Survey reveal most Australians fell short of usually meeting their recommended minimum number of serves from any of the FFGs [8] and during that period at least 35% of the total daily energy intake of adults and at least 39% for children was derived from discretionary foods [8]. Given that in Australia 63% of adults and 25% of children are overweight or obese [9], it is understandable why the ADGs state,

*There is limited capacity for including energy-dense discretionary foods in nutritious dietary patterns within the energy requirements of many Australians*.

[6] (p. 67)

Then in mid-2014 the Australian Commonwealth Government introduced a Health Star Rating (HSR) system as a voluntary and interpretive NBFOPL scheme for packaged, manufactured and processed foods. The system was developed in partnership with certain food industry, consumer and public health groups [10]. An algorithm assigns the number of stars between 0.5 and 5 that are attributed to a food based on so-called “positive nutrients” (protein, dietary fibre and any aspect of fruit, vegetable, legume or nut content) and so-called “risk nutrients” (sodium, sugars, saturated fat and total kilojoules) under the overarching objective, “*The more stars, the healthier the choice*” [11], although the advice to consumers is that it should be used to compare similar food products [12].

Evaluations of the HSR system’s implementation mostly have reported broadly positive findings for labelling implementation and consistency with the HSR system Style Guide [13], consumer awareness, understanding and use [13,14,15,16,17] and nutrient status [13]. Though another study reported that it has been a failure with consumers struggling to use it appropriately [18] albeit with a different colored background to the standard HSR format. A government commissioned independent report on submissions to a mid-2017 public consultation seeking stakeholder feedback on the merits of the HSR system has reported that most believed that the HSR has the potential to be a successful public health intervention, however many were critical about irregularities with the system such as a perception that HSRs don’t always align with the ADGs. Critically, the report states,

*When asked how effective the implementation of the HSR system has been in meeting the overall objectives, 12% of respondents indicated that the HSR is satisfactory or very effective, with the majority (73%) nominating unsatisfactory or ineffective*.

[19] (p. 7)

Although there have been some modelling studies, there has been no analysis of the HSR system’s actual implementation for its alignment with the ADGs. This is in spite of the Australia and New Zealand Food Regulation Ministerial Council having issued a policy statement on front of pack labelling that emphasised the need for such schemes to complement the ADGs [20]. Moreover, theoretical insights into food labelling posited by Caswell and Padberg [21], drawing on classical economic thinking, point out that the food label is more than a mechanism for conveying information. They apply these insights to propose that food labels have potential additional benefits in their ability to influence consumers’ decision-making and encourage manufacturers to implement guidelines.

This study aimed to investigate whether the HSR aligns with the ADGs and hence whether it supports or undermines them. Because the HSR system is one component of a broader ‘Healthy Food Partnership’ and within that context it is identified as providing an incentive for industry to reformulate their foods [22], we focussed our investigation on all new food products that entered the Australian marketplace following the launch of the HSR system.

## 2. Materials and Methods

### 2.1. Data Collection

Systematic sampling of all new Australian and New Zealand food and beverage product launches displaying a HSR using the Mintel Global New Products Database (GNPD) between 27 June 2014 (the date that the Australia and New Zealand Ministerial Forum on Food Regulation endorsed the HSR system [23] and 30 June 2017 was conducted. Mintel GNPD shoppers are trained to find new products when packaging indicate the product: is a re-launch, new formulation, new product, and/or new variety/extension; and/or new packaging can be recognised from the average shopper’s perspective. One researcher (SD) visually examined all food product labels displayed in the database to check for the presence of a HSR. Detailed information on all products was extracted, including the number of health stars displayed, GNPD food category and sub-category, release date, product description, packaging images, nutrition composition, and ingredients list.

### 2.2. Data Analysis

The food categories were based on those depicted in the 2013 ADGs with classifications informed by descriptions provided in the 2013 ADGs Educator Guide [6,7]. The ADG coding categories used were: FFG foods (fruit; vegetables; grain foods; meat/eggs/tofu/nuts/seeds/legumes; milk/yoghurt/cheese/alternatives; and mixed meals consisting mostly of FFG foods), Discretionary foods, and a small number of ‘other’ foods (culinary ingredients; formulated supplementary foods; and water).

The principles utilised by the Australian Bureau of Statistics (ABS) in its analysis of the 2011–2012 Australian Health Survey (AHS) were employed as the primary classification method for identifying discretionary foods. This included two aspects: the ABS’s Principles for Identifying Discretionary Foods, as detailed in the AHS User’s Guide; and the Discretionary Food List, wherein items from the ‘AUSNUT’ 2011–2013 food composition database are flagged as discretionary [24]. A transparent and documented procedure was created for products difficult to classify using the ABS method by consulting the ADGs [6]. Where there were difficulties in coding, each co-researcher attempted to code individually, and then all 3 reached a consensus decision based on the ingredients list and food purpose. A co-researcher (J.L.W.) undertook a validation procedure in which the coding of a random 5% of the sample was cross checked.

The HSR ranges and their rationale to analyse consistency of the food ratings with the ADGs were:

(i) Five food group foods (2.5–5 stars). The authors’ rationale for setting a minimum 2.5 stars (a ‘pass’ rating) for all FFG foods is that the ADG review process obtained epidemiological evidence that these foods were associated with healthy dietary patterns as reflected in the ADG advice to “*enjoy a wide variety of nutritious Five Food Group foods*” [6].
(ii) Discretionary foods: ‘compromise target’ (0.5–2 stars). The authors’ rationale for setting a maximum 2 stars (a ‘fail’ rating) for all discretionary foods is that the ADG review process obtained epidemiological evidence that these foods were associated with unhealthy dietary patterns as reflected in the ADG advice to *“limit intake of non-nutritious ‘discretionary’ foods”* [6].
  In addition, this maximum level is clearly below the minimum level of the range set for FFG foods to avoid any rating overlap that otherwise would obscure the necessary rating demarcation between the two categories.

Health star ratings on FFG and discretionary foods that fell outside of these ranges were classified as anomalies along with FFG food HSRs that were inconsistent with the intent of ADG qualified advice, e.g., general advice on ‘whole’ foods and/or specific advice about relative desirability, serve size and/or frequency of consumption. These product rating anomalies were analysed to identify the characteristics that explain their inconsistencies with the ADGs. Each anomaly is accompanied with one example from the data collected for this study.

### 2.3. Statistical Analysis

All statistical analyses were conducted in IBM SPSS Statistics version 23 (IBM, St Leonards, NSW, Australia). The frequency of products in the sample was compared to the frequency of products in the Mintel database during the same 3-year period. The frequency, median, minimum and maximum and interquartile range (IQR) for HSRs were produced for the total sample for: the ADG categories; and the ADG food categories collapsed into three groups, FFG, discretionary (including 2 frequent sub groupings), and other foods. Mann Whitney *U* tests were performed to determine any significant differences in median HSRs between FFG and discretionary foods.

## 3. Results

Of the 12,108 new products recorded on the Mintel GNPD during the 3-year data collection period, 1269 products displayed a HSR, representing 10.5% of all new products. This sample captured approximately 20% of all products carrying a HSR as figures from April 2017 indicated that more than 7000 products were displaying the HSR graphic in Australia [25]. The majority of the sample was FFG foods (57.2%), and of these, grains were represented at the highest frequency (27%), and dairy products at the lowest frequency (6.6%) (Table 1). Four food subcategories (breakfast cereals, meals and meal centres, processed fish/meat or chicken and snacks) made up more than half (53.8%) of all FFG foods displaying a HSR. Discretionary foods comprised 41.3% of the sample. Nearly half (47.5%) of discretionary foods were from the bakery and snack subcategories (Table 1).

The mean ranking of FFG foods (median 4.0) was significantly higher than the mean ranking of discretionary foods (median 2.5) (*p* < 0.05). Frequencies indicate 96.6% of FFG foods and 56.7% of discretionary foods received a HSR ≥ 2.5. Both FFG and discretionary foods had HSRs ranging from 0.5 to 5 stars, though the variability of discretionary foods (IQR 2.1) was higher than that for FFG foods (IQR 1). The median HSR for the categories of FFG foods ranged from 3.5 to 4.5. The median for discretionary snacks was higher at 4 compared to that of bakery foods (1.5) (Table 1). Formulated supplementary foods had a HSR of 4.5 or 5 and all culinary ingredients and waters had a HSR ≥ 2.5.

**Figure 1:**
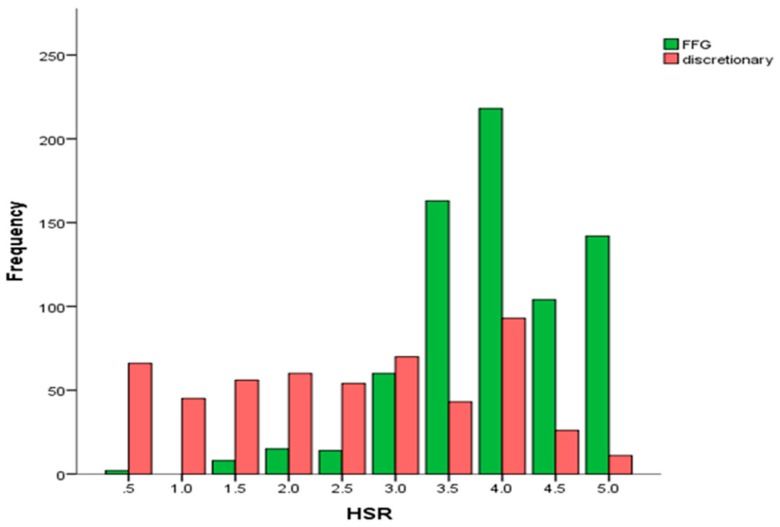
Frequency of Health Star Ratings (HSR) for five food group and discretionary foods.

A HSR of 4 stars was displayed most frequently on 25% of products and a HSR of 1 star least frequently on 3.5%, with an overall median of 3.5 stars. Figure 1 illustrates the frequency distribution of HSRs for FFG and Discretionary foods. It is clear that while FFG foods are skewed towards the higher end of the HSR scores (3.5–5), discretionary food HSRs are more spread out with the highest frequency at 4 stars and much overlap with FFG food HSRs.

A high number of product rating anomalies was identified. These anomalies arise most commonly because of inherent challenges with attempting to capture *food*-based recommendations using a *nutrient*-based scheme. Here we report on three types of common anomalies identified for the FFG food category and for the discretionary food category.

### 3.1. Five Food Group Category Anomalies

(i) Anomaly 1: Five food group foods displaying less than 2.5 stars
  An example of a FFG food displaying less than 2.5 stars is a smooth ricotta cheese displaying 1.5 stars [26].
(ii) Anomaly 2: Minimally processed, whole five food group foods displaying relatively modest HSRs and therefore inconsistent with the intent of ADG qualified advice
  An example of a minimally processed, whole FFG food displaying a relatively modest HSR (3 stars) is a raw, unsalted nuts product [27]. The ADG document advises that its recommendations are based on “whole foods”, and its Appendix G refers to concerns about the increasing consumption of processed foods [6]. Moreover, despite one intention of the HSR scheme being to create an incentive for foods to reformulate their composition in accordance with the ADGs, it is not physically possible for these foods to be reformulated and gain a higher score to display a higher number of stars.
(iii) Anomaly 3: Five food group foods displaying HSRs inconsistent with the intent of ADG qualified advice about its relative desirability, serve size and/or frequency of consumption
  An example of a FFG food displaying an HSR inconsistent with the intent of ADG qualified advice about its relative desirability, serve size and/or frequency of consumption is an apple juice displaying 5 stars [28]. The ADGs classify fruit juice as a FFG food [6] however the classification is accompanied by the qualification that “whole fruit is preferable to juice” and a 125 mL serve of fruit juice should be consumed “only occasionally”.

### 3.2. Discretionary Food Category Anomalies

(i) Anomaly 1: Discretionary foods displaying greater than 2 stars despite containing a significant amount of added sugar
  An example of a discretionary food displaying greater than 2 stars despite containing a significant amount of added sugar is a flavoured ice confection displaying 3 stars [29]. The ingredient list for the food product on which this relatively high HSR is being displayed indicates that added sugar contributes approximately 99% of the product’s energy content.
(ii) Anomaly 2: Discretionary foods displaying greater than 2 stars and containing substantial amounts of added nutrients/ingredients that increases ‘positive’ nutrient scoring
  An example of a discretionary food displaying and containing substantial amounts of added nutrients/ingredients that increases ‘positive’ nutrient scoring is a protein bar displaying 4 stars [30]. This product’s ingredient list indicates that it contains significant amounts of added whey protein isolate, milk protein concentrate, hydrolysed collagen and oligofructose. The HSR scheme’s algorithm calculates a significant number of positive points for the presence of these added protein and fibre ingredients, resulting in this discretionary food being eligible to display a relatively high HSR.
(iii) Anomaly 3: Discretionary foods displaying greater than 2 stars despite containing few nutrients
  An example of a discretionary food displaying greater than 2 stars despite its ingredient list indicating it contains few nutrients is a recipe base displaying 4 stars [31].

## 4. Discussion

The study results show a modest uptake of the HSR system amongst new products including a greater proportion of FFG foods over discretionary foods and with scores more concentrated at the higher end of the HSR distribution. This is consistent with the results of the two-year review of the HSR system [13]. It is unsurprising that foods most likely to score higher stars are participating more frequently in a voluntary scheme.

The majority of the total sample displayed a HSR ≥ 2.5, a finding similar to that reported elsewhere [32]. Worryingly, 56.7% of discretionary foods displayed a HSR ≥ 2.5. Although the median HSR for FFG foods (4 stars) was significantly higher than that of discretionary foods (2.5 stars) (*p* < 0.05), substantial overlap was observed. Both categories displayed HSRs ranging from 0.5 to 5, indicating some FFG foods are receiving HSRs too low and some discretionary foods are receiving HSRs too high to be consistent with the ADGs. A similar distribution of HSRs was observed in modelling studies that assessed alignment with ADGs [33,34,35].

For each of these modelling studies, HSRs for all packaged food and beverage items available in The George Institute for Global Health’s Australian food composition databases were calculated using packaging information collected in different years. Carrad et al. calculated HSRs on 20,225 products and concluded the HSR system categorised products in a way that reflected ADG’s recommendations, based on median star ratings being significantly higher for ‘core’ (their term) foods at 4 stars compared to discretionary products at 2 stars [33]. Anomalies in the HSR’s consistency however were recorded; ninety products classified as discretionary scored a five star rating, including sugar-free confectionary and protein bars.

Using a HSR of 3.5–5/5 stars (determined from concordance with “Green” traffic light criteria) to indicate consistency with FFG foods and a HSR of 0.5–3/5 stars to indicate discretionary foods, work commissioned by New South Wales Health examined appropriateness of the HSR system for use in school canteens and applied the HSR algorithm to 11,500 products across 30 food categories. Findings included a statistically significant difference in the mean HSR of FFG and discretionary foods (mean HSR 3.7 vs. 1.9), and that 79% of FFG groups received a HSR of ≥3.5, whereas 86% of discretionary foods scored below this cut-off.

Applying the algorithm to a large database of 34,135 packaged products, Peters et al. found the HSR could discriminate between core and discretionary foods on the basis of differences in the median HSR scores, however discrimination was improved when added sugars instead of total sugars were used in the algorithm [35]. Similar to the other studies the median HSR for ‘core’ (their term) foods was 4 stars and the median for discretionary foods was 2 stars, yet substantial overlap of these food groups was observed for all food categories, with between 5–40% of discretionary foods (depending on the food category) scoring an HSR ≥ 4.

In addition, a study conducted by the Cancer Council calculated HSRs for 1363 FFG dairy products from four large supermarkets, concluded the HSR system was consistent with ADGs based on lower-fat products receiving higher star ratings [36].

The main conclusion of all of these modelling studies is that the HSR is consistent with the ADGs based on the median HSR for FFG foods being statistically higher than the HSR for discretionary foods. However, there are a number of questions to be raised about the robustness of this conclusion. The cut-points for accepting foods as ‘core’ (their term) or discretionary in these studies seem particularly arbitrary and this can significantly affect conclusions as arises when discretionary foods displaying an HSR of 3 stars are considered to be aligned with the ADGs. There is limited consideration given to the relatively high proportion of foods, in particular discretionary foods, which display a high HSR. The relatively smaller proportion of discretionary foods with high HSRs in the above studies compared to the present study may be as a result of applying the HSR in modelling studies based on previous food surveys rather than assessing the reality of foods displaying the HSR in the marketplace. In all of these studies, the HSR scores have been estimated since values for certain components of the HSR calculator are not always available on the food label and this could result in inaccurate scores for some foods. The NSW Health commissioned study also excluded a large number of foods, many of which would be classified as discretionary, and it is difficult to determine the impact of these exclusions.

There are HSR scheme governance features as well as algorithm technical and design flaws that help explain the present study’s findings. The HSR development, implementation and evaluation is governed as a partnership with government, non-government organisations and food industry. The HSR Advisory Committee, appointed by Food Ministers, includes representatives with commercial conflicts of interest. Conversely, no public health nutritionist with experience in and detailed knowledge of the development of the ADGs and their underlying evidence-base was appointed to the Committee or the associated HSR Technical Advisory Group. The literature consistently reports that industry involvement in policy/program development contributes to consumer misinformation, undermines the integrity of the system, consumer trust and the ability to achieve and protect public health outcomes [37,38,39]. This situation is complicated by the lack of transparency about how and why decisions were originally made with the design of the HSR algorithm.

The potential influence of conflicted interests raises questions about the decision-making processes that resulted in the HSR system being voluntary and positively oriented. These two HSR system characteristics facilitate beneficial outcomes for commercial interests over public health interests. The voluntary nature of the HSR system, as with all NBFOPL schemes, means that food manufacturers can pick and choose which of and when their products display HSRs. The relatively low number of food products with less than 2.5 stars, high number with 2.5 stars and above and overall high median are suggestive of a selective positive bias in the HSR implementation. The positive orientation of the HSR system means that all food products get ‘health’ stars and benefit from a possible ‘halo’ effect to support their marketing irrespective of whether they are a FFG or discretionary food.

There are technical flaws with the algorithm that adversely affect HSRs for FFG and discretionary foods and help explain many of the observed anomalies. In relation to FFG food anomalies, the algorithm has calculated low HSRs for creamed ricotta and minimally processed, unsalted macadamia nuts apparently due to their saturated fat content. This is despite foods being more than the sum of the individual nutrients they contain. Within a food there are synergies among nutrients and the complexity of the food matrix structure affects the absorption and availability of nutrients. Therefore single nutrients such as saturated fat are not reliable predictors of the relationship between any single food and non-communicable diseases. As Thorning et al. comment in the case of dairy food,

*The food matrix may exhibit a different relation with health indicators compared to single nutrients studied in isolation. … the nutritional values of dairy products should not be considered on the basis of the biofunctionality of the nutrients within dairy structures*.

[40] (p. 1033)

And this is borne out by the evidence statements that accompanied the ADGs reporting that all cheese was associated with less cardiovascular disease (CVD) and stroke risk (Grade B evidence), less type 2 diabetes risk and not associated with weight change or risk of obesity (Grade C evidence) and consumption of nuts does not lead to weight gain in the short-term and reduces cholesterol levels (Grade C evidence) [41]. An inherent limitation with nutrient-based schemes is that they have limited ability to capture qualified advice that might accompany food-based recommendations. This limitation was demonstrated with the anomaly that arose with apple juice displaying 5 stars despite the ADG recommendation being qualified with advice that whole fruit is preferable, and juice should be consumed only occasionally.

In relation to discretionary food anomalies, the algorithm has failed to adequately penalise the presence of added sugar in foods resulting in a number of high added sugar containing discretionary foods avoiding low HSRs. This is despite data from the 2011–2012 AHS indicating most Australians are far exceeding maximum added sugar recommendations, making this matter ripe for reconsideration [42]. Conversely, another technical flaw in the algorithm is resulting in some food products gaining positive points from sources such as added protein isolates/fibre/fruit concentrates resulting in a higher HSR than otherwise might be anticipated. The anomaly highlighting that a protein bar product was displaying 4 stars illustrates this situation as the presence of significant amounts of protein isolates generated positive points. This is despite data from the AHS indicating that Australians already consume a sufficient amount of protein, creating little public health benefit from incentivising further uptake [9]. The anomaly illustrating that a recipe mix could display 4 stars despite not being an inherently nutritious food is due to the premise that the product would be prepared with another food (lamb/vegetables) that in combination would elevate its HSR. The ability to elevate points for one product based on points that might accrue from the presence of another product is a form of technical loophole in the HSR system’s Style Guide advice, rather than a technical flaw in the algorithm per se.

In addition there are design flaws with the algorithm. The HSR system has failed to adequately integrate nutrition science principles into the planning and design of the algorithm to support its alignment with the ADG recommendations. For example, a core message of the ADGs is to eat more FFG foods and less discretionary foods. Yet, this distinction is difficult to put into practice when the tool being used to inform food labelling abstracts nutrients from their source food. In other words the algorithm has limited capacity to distinguish whether a nutrient that it is scoring is derived from a nutritious FFG food or an unhealthy discretionary food.

Although it is intuitively appealing to focus on individual nutrients to prevent obesity and NCDs, this is not always borne out by the evidence derived from food and dietary pattern studies. The evidence consistently shows that it is nutrient and food combinations in the overall diet profile that is predictive of health outcomes rather than attempting to ascribe a ‘healthiness’ score to individual foods to predict those outcomes. The scientific methods and evidence bases that inform the ADGs and the HSR system differ. The nutrition science used to inform the revised ADGs was of high quality. Evidence was synthesised from six major sources of scientific information: the existing ADG knowledge base; the Nutrient Reference Values; dietary modelling of composite food groups to identify serving sizes and minimum number of serves required to meet nutritional needs in Australia; systematic, graded, literature reviews of over 55,000 studies on the links between foods/nutrients and health outcomes; current food and nutrient intakes and dietary patterns; and key authoritative reports.

In comparison, the nutrition science used to inform the HSR system’s nutrient profiling approach is of low quality. The science used by the HSR system categorises the ‘healthiness’ of individual foods based on their content of a small number of selected nutrients for which arbitrary cut-off levels are set and not by systematic review or public consultation. Evidence will vary with how the nutrient profiling method is designed and implemented and assumptions about which nutrients and which cut off levels are fed into the nutrient profiling [36]. Although algorithms might be powerful for mathematical modelling they are less able to accurately simulate the complex mechanisms by which nutrients interact and behave within foods to predict their relationships with obesity and NCD outcomes. There is no evidence that the nutrient profiling and algorithm that underpins the HSR system helps prevent obesity or NCDs.

FBDGs are based on the nutrition science principle that *“Foods make up diets; foods are more than just a collection of nutrients”* [43]. Therefore it is simplistic for the HSR scheme to abstract individual nutrients from foods and suggest that awarding points on the basis of so-called ‘positive’ and ‘risk’ nutrients in a food can accurately or adequately represent complex dietary patterns. This is a core reason why nutrition science is moving away from nutrient-specific approaches to more holistic approaches examining food and dietary patterns approaches for obesity and NCD prevention [44,45,46].

### 4.1. Outcomes

These findings provide evidence that the HSR system is unlikely to be effective in helping prevent obesity and NCDs and indeed may be counterproductive in this regard. The data do indicate that a significant difference exists between the median star ratings for FFG and discretionary foods and the system works reasonably well for rating FFG foods with this category’s median star rating (4 stars) being in accordance with ADG recommendations (with some notable anomalies). However, in spite of the significant difference in medians, the scheme is working very poorly for rating discretionary foods with this category’s median star rating (2.5 stars), and 56.7% of such products displaying ≥2.5 stars. Effectively the scheme is creating a health halo effect and supporting the marketing of many discretionary foods in direct contradiction of ADG recommendations. This situation is especially problematic with the majority of the Australian population needing to consume more FFG foods and less discretionary foods. In this context it is incongruous to have a public health scheme that promotes the use of ‘health’ stars on discretionary foods let alone with a median of 2.5 stars.

A paper by Ni Mhurchu et al. reports that the system is fostering modest reformulation towards less saturated fat, salt and energy in certain food products and the authors argue that this will benefit public health [32]. The authors do not specify whether the reformulation is occurring for FFG, discretionary foods or both. This specification is especially relevant in context of positively-oriented NBFOPL schemes, such as the HSR, because if reformulation is rewarded with higher HSR ratings it can create a marketing opportunity for discretionary foods and be a risk for public health. Focussing on individual nutrients in isolation of a food context can introduce contradictions with broader nutrition objectives. For instance, the presence of low-nutrient claims on food products does not necessarily correlate with the overall nutritional quality of the product relative to products not displaying claims [47] and ignores the basic nutrition objective of promoting healthy food selection for nutrient adequacy.

In broader terms there is an opportunity cost forgone with the HSR system. Currently it is the only nutrition activity receiving sizeable Commonwealth government investment. It appears to be diverting government attention and resources away from priority issues such as the development of a national nutrition policy. Indeed, it has been used as a reason not to have certain strong policy schemes, e.g., when asked in early 2017 about the government’s intention to introduce a sugar tax, the Assistant Health Minister replied that it was unnecessary because,

*We’ve also got running a collaborative called the healthy food partnership, we are driving that with the health star ratings*.

[48]

At a more fundamental level, the HSR system is contributing to a shift in power in Australia over the public health nutrition agenda and who is in control of this agenda. It is re-framing the agenda away from a food and dietary pattern paradigm towards a nutrient paradigm that focuses on the labelling and reformulation of discretionary foods as the solution to nutrition problems. And it is empowering certain stakeholders with commercial interests that may benefit from such labelling and reformulation to be directly engaged with the decision-making for such activities.

### 4.2. Strengths and Limitations

This study was the first to systematically and comprehensively analyse all new foods entering the Australian marketplace displaying HSRs for their alignment with the ADGs. The research design was timely given the relatively recent availability of the national FBDGs and NBFOPL scheme and it was novel in its ability to access a database of food labels that is comprehensive and up-to-date as well as a classification system for demarcating FFG and discretionary foods.

However, the findings need to be interpreted in accordance with a number of data collection and analysis constraints. The Mintel GNPD may not necessarily capture all foods in the marketplace displaying an HSR symbol because data is only collected for new products and so the addition of an HSR symbol onto the label of a pre-existing food would not be recognised. In addition, the data relate to the number of products displaying an HSR symbol and not the products’ market share. Therefore, the data cannot be used to infer the proportion of market sales or dietary intakes affected by the HSR system. The sample size is modest relative to the total number of products displaying the HSR. However, the modest size reflects the study’s aim in which we recognized that because the HSR was a new labelling concept, we believed it important to investigate how it is being used particularly on new food products, as an indication of how manufacturers are responding to its introduction. In this regard, the Mintel GNPD is an especially relevant database because it comprehensively records product innovation and new product activity in the marketplace. The substantial number and rapidly evolving nature of novel and mixed foods in the marketplace presented challenges to the ability of the ABS classification scheme and ADG Educator’s Guide to accurately classify all food products. The current ABS classification scheme itself was determined by expert opinion and potential anomalies with certain foods are inevitable and resources need to be invested into further developing the scheme to strengthen the principles for classifying FFG and discretionary foods.

Into the future there are two particular priority research agendas that follow on from this study. First, is the need to evaluate the ADG alignment of all foods displaying HSRs. This research would require supplementing the Mintel database with those pre-existing foods that are not recorded in the database but nonetheless change their label to include HSRs. Second, there is increasing interest in the importance of promoting healthy and sustainable food systems for protecting food and nutrition security as well as tackling dietary excess and imbalances [49]. The specification and measurement of criteria for food system recommendations extend beyond those for NBFOPL schemes and FBDGs. Just as this study has aimed to analyse the alignment of the HSR system with the ADGs, a future research priority would be to analyse the alignment of the HSR system and ADGs with this progressive food system agenda and recommendations.

### 4.3. Strengthening the Alignment of the Health Star Rating System with the Australian Dietary Guidelines

(i) Amend the HSR system’s governance arrangements

Two particular amendments to the HSR system’s governance arrangements will strengthen consumer trust in the system and assist with the achievement of the algorithm technical adjustments and design reforms that are necessary for better alignment with the ADGs. First, there is a need for improved transparency of decision-making processes, e.g., how and why certain nutrients are selected and their cut off levels are set within the algorithm. Second, the memberships of the HSR Advisory Committee and Technical Advisory Group need reviewing to reduce real and/or perceived conflicts of interest and increase public health nutrition expertise and experience with the ADGs.

(ii) Adjust the HSR algorithm’s technical flaws

- All minimally processed, whole FFG foods to be eligible to display five health stars
- Definitions for nutrients and ingredients which positively contribute to HSR scores need to exclude the addition of ingredients with negligible health benefit. For example, preventing the inclusion of soy isolate for protein points and preventing fruit juice concentrates being added to discretionary foods to garner extra HSRs.
- Increase penalties for ‘added’ sugar and remove penalties for naturally occurring sugar (lactose, fructose) present in FFG foods. One study has reported that reforming the HSR algorithm to include added sugar will improve discrimination between FFG and discretionary foods, though the criteria for classifying FFG and discretionary foods was not explained [35].
- Amend the HSR Style Guide to close the ‘as prepared’ loop hole so that the HSR is calculated on the food ‘as sold’ and not on a manufacturer’s claim about how it might be prepared with other foods and/or ingredients.

(iii) Reform the HSR algorithm design flaws

A core challenge for aligning a NBFOPL scheme with FBDG recommendations is how to ensure a nutrient-oriented construct can capture the evidence and concepts of a food/diet-oriented construct. Overlaying a nutrient-oriented algorithm with food/diet-oriented recommendations to demarcate nutritious foods from non-nutritious foods provides one approach to address this challenge. For the HSR system this might be achieved by capping the minimum and maximum number of health stars that can be displayed on FFG and discretionary foods respectively, e.g., a minimum 2.5 stars for FFG foods and a maximum 2 stars for discretionary foods. The demarcation could be further highlighted by color coding the HSR symbol for FFG and discretionary foods green and red respectively. However, this approach still means that discretionary foods are eligible to be marketed with HSRs, an outcome at odds with the ADGs. Therefore an approach more aligned with the ADGs would require that eligibility to display positive (green-colored) health stars being restricted to FFG foods and instead (red-colored) warning-type symbols being substituted for ‘health’ stars on discretionary foods; an approach similar to the Chilean mandatory front-of-pack warning label for foods high in calories, sugars, sodium or saturated fat [50]. In both situations a graded scale could be retained to provide an incentive for reformulation either to increase the number of health stars on FFG foods or to reduce the number of warning symbols displayed on the labels of discretionary foods.

With its current flaws and large proportion of discretionary foods displaying 2.5 stars or above it would be counterproductive for the HSR system to be mandatory. However, once the adjustments to the algorithm’s technical flaws and the reforms of its design flaws have been implemented the HSR system then should be mandated. Mandating the HSR system at that time would increase the system’s implementation providing for a more comprehensive and unbiased comparison of the HSR across foods because all labels would be required to display an HSR regardless of whether it be a relatively high or low number of stars.

## 5. Conclusions

The HSR system is undermining the ADG recommendations. Nutrient-based HSRs displayed on a substantial proportion of foods are non-concordant with food-based advice to increase consumption of FFG foods and reduce consumption of discretionary foods. The HSR system is contributing to a confusing food and nutrition information environment, potentially exacerbating prevalent dietary excesses and imbalances and creating a public health risk. The flaws with the HSR system are an inevitable consequence of misrepresenting nutrition science and expecting reductionist (nutrient) schemes to be sufficient to solve nutrition problems that are primarily holistic (dietary excesses and imbalances) in their causation.

Amending the HSR system’s governance arrangements and adjusting its algorithm to correct its technical flaws will help strengthen consumer trust in the system and reduce the number of HSR anomalies. However, the undermining of ADG recommendations primarily is the consequence of a design flaw with the algorithm failing to incorporate a food and dietary pattern context into the nutrient profiling that underpins the HSR system. Non-concordant rating outcomes are systemic problems that will persist if nutrient-oriented schemes are not aligned with food/diet-oriented reference standards. This has universal relevance for planning NBFOPL schemes regardless of national circumstances. The solution is for nutrient profiling techniques to incorporate within their algorithms criteria that capture the evidence from food/diet and health research to demarcate nutritious and non-nutritious foods.

Expectations about the HSR system and NBFOPL schemes in general need to be kept in perspective. Even when well designed and appropriately informed by nutrition science, NBFOPL schemes can only achieve so much in tackling dietary risk factors. Comprehensive all-of-government nutrition policies are essential if society is to effectively and safely prevent obesity and NCDs. Frustratingly, in Australia much investment of time and budget as well as political and professional will have been directed at reacting to the flawed HSR system and this appears to have been at the expense of realising a national nutrition policy. There is a potential constructive role for a reformed HSR system to contribute as one modest component within such a national nutrition policy in the future but first that policy needs to be formulated and implemented.

## Figures and Tables

**Table 1:** Frequency and median Health Star Ratings (HSR) for Australian Dietary Guidelines (ADG) and other food groups.

ADG Food Group	*n* (%)	HSR Median	Min, Max	IQR	*n* HSR ≤ 2	*n* HSR ≥ 2.5
**FFG Foods**	**726 (57.2)**	**4**	**0.5, 5**	**1**	25 (3.4% *)	701 (96.6% *)
Grains	195 (27) *	4	1.5, 5	1.0	6	189
Fruit	113 (15.6) *	4.5	1.5, 5	1.0	1	112
Vegetables	100 (13.8) *	4.5	3, 5	1.3	0	100
Meat/legumes/nuts/seeds/eggs	150 (20.7) *	4	1.5, 5	1.0	10	140
Dairy/alternatives	48 (6.6) *	4	0.5, 5	1.5	6	42
Mixed meals	120 (16.5) *	3.5	2, 5	0	2	118
**Discretionary Foods**	**524 (41.3)**	**2.5 †**	**0.5, 5**	**2.1**	227 (43.3% *)	297 (56.7% *)
Snacks	143 (27.3) *	4	1, 5	1.0	28	115
Bakery	107 (20.3) *	1.5	0.5, 4	2.0	76	31
**Other Foods**	**19 (1.5)**	**4.5**	**3, 5**	**1.0**	**0 (0%)**	**19 (100%)**
Culinary	8 (0.6)	4	3, 4	1	0	8
Formulated Supplementary Foods	6 (0.5)	4.5	4.5, 5	0.1	0	6
Water	5 (0.4)	5	5, 5	-	0	5
**Total Sample**	**1269**	**3.5**	**0.5, 5**	**1.5**	**277**	**992**

*n* = number of food products; IQR = interquartile range; FFG = five food group. † HSR mean rank significantly different to FFG mean rank (*p* < 0.05), Mann Whitney *U* test; * % of products within food grouping.

## References

[1] GBD 2016 Risk Factor Collaborators (2017). Global, regional, and national comparative risk assessment of 84 behavioural, environmental and occupational, and metabolic risks or clusters of risks, 1990–2016: A systematic analysis for the Global Burden of Disease Study 2016. Lancet.

[2] Food-Based Dietary Guidelines. http://www.fao.org/nutrition/education/food-dietary-guidelines/en/.

[3] Campos S., Doxey J., Hammond D. (2011). Nutrition labels on pre-packaged foods: A systematic review. Public Health Nutr..

[4] Lawrence M. (2017). Rethinking the translation of nutrition evidence into public health practice. J. Nutr. Intermed. Metab..

[5] Rayner M. (2017). Nutrient profiling for regulatory purposes. Proc. Nutr. Soc..

[6] National Health and Medical Research Council (2013). Australian Dietary Guidelines.

[7] National Health and Medical Research Council (2013). Australian Dietary Guidelines Educator Guide.

[8] Australian Bureau of Statistics 4364.0.55.012-Australian Health Survey: Consumption of Food Groups from the Australian Dietary Guidelines, 2011–2012. http://www.abs.gov.au/ausstats/abs@.nsf/Lookup/4364.0.55.012main+features12011-12.

[9] Australian Bureau of Statistics (2014). Australian Health Survey: Nutrition First Results-Foods and Nutrients, 2011–2012.

[10] Health Star Rating System. http://healthstarrating.gov.au/internet/healthstarrating/publishing.nsf/content/home.

[11] About Health Star Ratings. http://healthstarrating.gov.au/internet/healthstarrating/publishing.nsf/Content/About-health-stars.

[12] How to Use Health Stars. http://www.healthstarrating.gov.au/internet/healthstarrating/publishing.nsf/Content/How-to-use-health-stars.

[13] Health Star Rating Advisory Committee (2017). Two Year Progress Review Report on The Implementation of the Health Star Rating System.

[14] Pettigrew S., Talati Z., Miller C., Dixon H., Kelly B., Ball K. (2017). The types and aspects of front-of-pack food labelling schemes preferred by adults and children. Appetite.

[15] Talati Z., Pettigrew S., Dixon H., Neal B., Ball K., Hughes C. (2016). Do health claims and front-of-pack labels lead to a positivity bias in unhealthy foods?. Nutrients.

[16] Talati Z., Pettigrew S., Hughes C., Dixon H., Kelly B., Ball K., Miller C. (2016). The combined effect of front-of-pack nutrition labels and health claims on consumers’ evaluation of food products. Food Qual. Prefer..

[17] Talati Z., Pettigrew S., Kelly B., Ball K., Dixon H., Shilton T. (2016). Consumers’ responses to front-of-pack labels that vary by interpretive content. Appetite.

[18] Hamlin R., McNeill L. (2016). Does the Australasian “Health Star Rating” front of pack nutritional label system work?. Nutrients.

[19] MP Consulting (2017). Report on Submissions to the Five Year Review of the Health Star Rating System.

[20] Australia and New Zealand Food Regulation Ministerial Council (2009). Front of Pack Labelling Policy Statement.

[21] Caswell J.A., Padberg D.I. (1992). Toward a more comprehensive theory of food labels. Am. J. Agric. Econ..

[22] About the Partnership. http://www.health.gov.au/internet/main/publishing.nsf/Content/about-the-partnership.

[23] Front-of-Pack Labelling Updates. http://foodregulation.gov.au/internet/fr/publishing.nsf/Content/front-of-pack-labelling-1.

[24] Australian Bureau of Statistics 4363.0.55.001-Australian Health Survey: Users’ Guide, 2011–2013. http://www.abs.gov.au/ausstats/abs@.nsf/Lookup/4363.0.55.001Chapter65062011-13.

[25] Australia and New Zealand Ministerial Forum on Food Regulation Communiqué 28 April 2017. http://foodregulation.gov.au/internet/fr/publishing.nsf/Content/forum-communique-2017-April.

[26] Coles Supermarkets Australia Pty Ltd.. https://shop.coles.com.au/a/a-national/product/coles-brand-smooth-ricotta-375g.

[27] Woolworths. https://www.woolworths.com.au/shop/productdetails/231808/macro-nuts-macadamia-kernels.

[28] The Daily Juice Company. http://www.thedailydrinksco.com/daily-juice/product-apple.

[29] (1662). Woolworths. https://www.woolworths.com.au/shop/productdetails/21662/zooper-dooper-8-cosmic-flavours.

[30] Smart Living Nutrition. http://www.smartlivingnutrition.com.au/apple-cinnamon-love-me-low-carb/.

[31] Maggi. https://www.maggi.com.au/products/recipe-bases/lamb-casserole.

[32] Mhurchu C., Eyles H., Choi Y. (2017). Effects of a voluntary front-of-pack nutrition labelling system on packaged food reformulation: The Health Star Rating System in New Zealand. Nutrients.

[33] Carrad A.M., Louie J.C.Y., Yeatman H.R., Dunford E.K., Neal B.C., Flood V.M. (2016). A nutrient profiling assessment of packaged foods using two star-based front-of-pack labels. Public Health Nutr..

[34] Dunford E., Cobcroft M., Thomas M., Wu J. (2015). Technical Report: Alignment of New South Wales Healthy Food Provision Policy with the Health Star Rating System.

[35] Peters S., Dunford E., Jones A., Ni Mhurchu C., Crino M., Taylor F., Woodward M., Neal B. (2017). Incorporating added sugar improves the performance of the Health Star Rating Front-of-Pack Labelling System in Australia. Nutrients.

[36] Wellard L., Hughes C., Watson W.L. (2016). Investigating nutrient profiling and Health Star Ratings on core dairy products in Australia. Public Health Nutr..

[37] Mozaffarian D. (2017). Conflict of interest and the role of the food industry in nutrition research. JAMA.

[38] Moodie R., Stuckler D., Monteiro C., Sheron N., Neal B., Thamarangsi T., Lincoln P., Casswell S. (2013). Profits and pandemics: Prevention of harmful effects of tobacco, alcohol, and ultra-processed food and drink industries. Lancet.

[39] Brown K., Rundall P., Lobstein T., Mwatsana M., Jeffery B. (2017). Open letter to WHO DG candidates: Keep policy and priority setting free of commercial influence. Lancet.

[40] Thorning T.K., Bertram H.C., Bonjour J.-P., de Groot L., Dupont D., Feeney E., Ipsen R., Lecerf J.M., Mackie A., McKinley M.C. (2017). Whole dairy matrix or single nutrients in assessment of health effects: Current evidence and knowledge gaps. Am. J. Clin. Nutr..

[41] National Health and Medical Research Council (2011). A Review of the Evidence to Address Targeted Questions to Inform the Revision of the Australian Dietary Guidelines.

[42] Australian Bureau of Statistics (2016). Australian Health Survey: Consumption of Added Sugars 2011–2012.

[43] World Health Organization (1998). Preparation and Use of Food-Based Dietary Guidelines.

[44] Mozaffarian D., Ludwig D.S. (2010). Dietary guidelines in the 21st century—A time for food. JAMA.

[45] Stanton R. (2016). Changing eating patterns versus adding nutrients to processed foods: Food-based dietary guidelines are necessary but the processed food industry prefers to concentrate on individual nutrients. Med. J. Aust..

[46] Fardet A., Rock E. (2014). Toward a new philosophy of preventive nutrition: From a reductionist to a holistic paradigm to improve nutritional recommendations. Adv. Nutr..

[47] Taillie L.S., Ng S.W., Xue Y., Busey E., Harding M. (2017). No Fat, no sugar, no salt..., no problem? Prevalence of “low-content”; nutrient claims and their associations with the nutritional profile of food and beverage purchases in the United States. J. Acad. Nutr. Diet..

[48] ABC Radio National Breakfast with Fran Kelly. Mpegmedia.abc.net.au/rn/podcast/2017/02/bst_20170221.mp3.

[49] High Level Panel of Experts (2017). Nutrition and food systems. A Report by the High Level Panel of Experts on Food Security and Nutrition of the Committee on World Food Security. http://www.fao.org/fileadmin/user_upload/hlpe/hlpe_documents/HLPE_Reports/HLPE-Report-12_EN.pdf.

[50] Ramirez N. (2015). Chile’s New Nutritional Labeling Law.

